# A Model of Binocular Rivalry and Cross-orientation Suppression

**DOI:** 10.1371/journal.pcbi.1002991

**Published:** 2013-03-28

**Authors:** Christopher P. Said, David J. Heeger

**Affiliations:** Center for Neural Science and Department of Psychology, New York University, New York, New York, United States of America; Université Paris 5, and CNRS, France

## Abstract

Binocular rivalry and cross-orientation suppression are well-studied forms of competition in visual cortex, but models of these two types of competition are in tension with one another. Binocular rivalry occurs during the presentation of dichoptic grating stimuli, where two orthogonal gratings presented separately to the two eyes evoke strong alternations in perceptual dominance. Cross-orientation suppression occurs during the presentation of plaid stimuli, where the responses to a component grating presented to both eyes is weakened by the presence of a superimposed orthogonal grating. Conventional models of rivalry that rely on strong competition between orientation-selective neurons incorrectly predict rivalry between the components of plaids. Lowering the inhibitory weights in such models reduces rivalry for plaids, but also reduces it for dichoptic gratings. Using an exhaustive grid search, we show that this problem cannot be solved simply by adjusting the parameters of the model. Instead, we propose a robust class of models that rely on ocular opponency neurons, previously proposed as a mechanism for efficient stereo coding, to yield rivalry only for dichoptic gratings, not for plaids. This class of models reconciles models of binocular rivalry with the divisive normalization framework that has been used to explain cross-orientation. Our model makes novel predictions that we confirmed with psychophysical tests.

## Author Summary

Binocular rivalry is a visual illusion that occurs when the two eyes are presented with incompatible images. Instead of perceiving a mixture of the two images, most people tend to experiences alternations in which they only see one image at a time. Binocular rivalry is more than just an interesting illusion: it reflects actual competition between neurons in the brain, and therefore provides a rare window into neural dynamics. To help us understand these mechanisms, researchers have developed several computational models of binocular rivalry. Yet surprisingly, as we show in this paper, previous computational models of rivalry make an incorrect prediction. They predict that certain types of images (similar to checkerboards) will cause strong perceptual alternations even when viewed normally. Since this prediction doesn't hold up, the existing models must not be telling the whole story. In this paper, we develop a new model of binocular rivalry that doesn't make this prediction. The model also makes novel predictions – not made by conventional models – that stand up to experimental test. Our model thus provides a better account of how neurons in the visual system interact with one another.

## Introduction

Binocular rivalry is a visual phenomenon in which perception alternates between incompatible monocular images presented to the two eyes [1]–[4]. For example, when one eye is presented with an oriented grating and the other eye is presented with an orthogonal grating, observers experience alternating periods of dominance in which one grating is visible and the other is invisible or nearly invisible.

Computational models have been proposed to characterize the alternating periods of perceptual dominance experienced during rivalry [5]–[14]. These models rely on mutual inhibition between neurons representing the two percepts, so that when one percept is dominant, the other percept is suppressed. To capture alternations between the percepts, rivalry models include endogenous noise, adaptation, or a combination of both.

**Figure 1:**
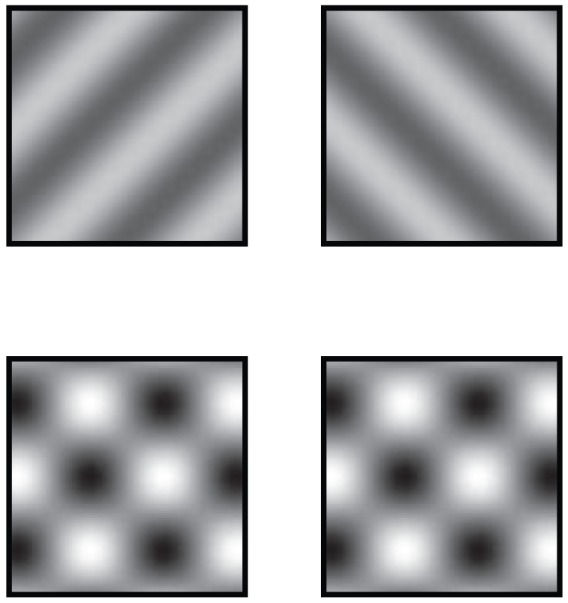
Demonstration of strong binocular rivalry and weak pattern rivalry. Although experiments on rivalry are typically done under controlled laboratory conditions with prisms or stereoscopes, some readers may be able to experience the effect by crossing their eyes, aligning the left and right boxes so that a total of six boxes are observed, rather than four. If done correctly, the top middle box will display dichoptic gratings, and observers experience strong rivalry, with clear alternations in dominance between leftward-oriented and rightward-oriented gratings. The bottom middle box will display binocular plaids, where each eye is shown the same two superimposed orthogonal components (leftward and rightward gratings), for which any alternations in the perceived strength of the components are very weak.

While these models successfully explain the case of dichoptic gratings, in which incompatible gratings are presented separately to the two eyes (Figure 1, *top*), the binocular rivalry literature has largely overlooked the case of binocular plaids, in which a pair of orthogonal gratings are presented superimposed to both eyes (Figure 1, *bottom*). Because models of binocular rivalry rely on strong competition between neurons tuned to orthogonal orientations, they typically predict rivalry between plaid components that is nearly as strong as the rivalry between dichoptic gratings. This prediction is not borne out by psychophysical evidence or by subjective experience. While plaid components show a type of rivalry known as ‘pattern rivalry’, it is far weaker than the rivalry experienced with dichoptic gratings [15], [16] (Figure 1). Instead of strong rivalry, plaid components undergo much weaker competition caused by “cross-orientation suppression”, in which the neural responses to each of the component gratings is lower than it would have been without superimposing the other component grating [17]–[19]. Cross-orientation suppression is well characterized by the normalization equation, according to which neural responses are normalized (i.e., divided) by a common factor, which includes the summed activity of a large pool of neurons [19], [20]. The normalization equation was developed to explain a variety of response properties of neurons in primary visual cortex, including cross-orientation suppression, surround suppression, and response saturation at high contrasts.

Ocular opponency neurons compute the difference in the signals between the two eyes, and have been identified using both neurophysiology [21]–[23] and psychophysics [24], [25]. Because of their potential to reduce redundancies between the eyes, they have been proposed as part of a theory of efficient stereo coding [26], a topic which might appear to be unrelated to rivalry and cross-orientation suppression.

In this paper, we explain how it is possible for dichoptic gratings to rival strongly while plaid components rival only weakly, and how to reconcile models of binocular rivalry with the normalization model. We propose a firing rate model that relies on ocular opponency neurons because they uniquely signal when rivalry should occur. For each orientation, opponency neurons receive excitation from one eye, and inhibition from the other eye. For binocular plaids, the opponency neurons in the model are silent because their inhibitory and excitatory inputs cancel. Under these circumstances, conventional normalization causes weak cross-orientation suppression. For dichoptic gratings, the opponency neurons are active, and we propose that they inhibit (through feedback) the monocular neurons corresponding to the eye from which they receive inhibition, thus amplifying the competition between the two eyes, resulting in rivalry. To account for the fact that binocular rivalry suppresses all orientations equally [27], feedback inhibition is directed towards all orientations. Opponency neurons may be the neurobiological analogue of rivalry XOR (exclusive-OR) units; These XOR units were proposed 23 years ago to prevent plaid component rivalry in a qualitative model of binocular rivalry [28], but have been overlooked ever since.

To test the theory, we performed a psychophysics experiment that investigated a novel prediction of the model. According to the model, rivalry is predicted to be weaker following adaptation with monocular stimuli than binocular stimuli. This prediction follows from the fact that monocular stimuli evoke strong responses (and hence adaptation) in the opponency neurons, whereas binocular stimuli do not. A conventional model without opponency neurons does not make this prediction. Our psychophysical results, along with previously published psychophysical results [29] (see Discussion), supported the prediction of the ocular opponency model.

## Results

### Conventional model

**Figure 2:**
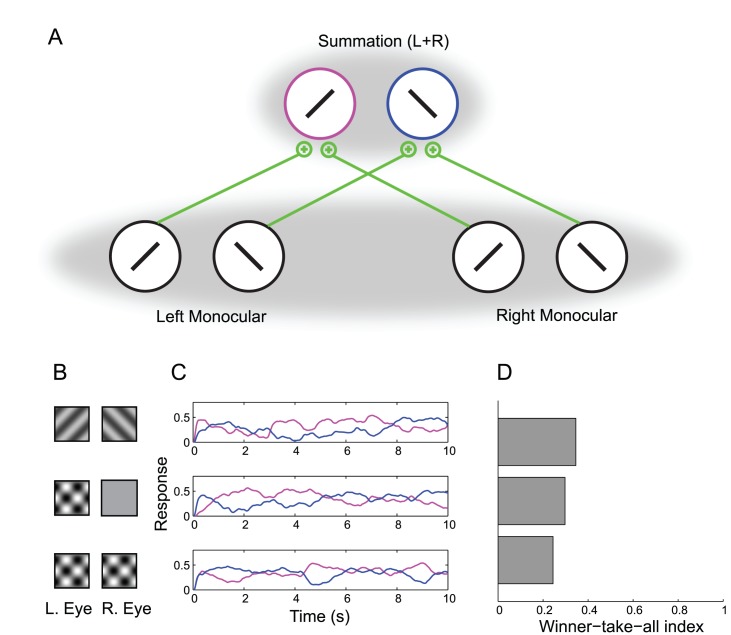
Conventional model. (A) Schematic. Monocular neurons drive iso-oriented binocular summation neurons with excitatory feedforward connections (green). Mutual inhibition within each layer is implemented by a normalization pool (gray shadows). (B–D) Model simulations. Top row: dichoptic gratings. Middle Row: monocular plaid. Bottom row: binocular plaids. (B) Stimulus conditions. (C) Example response time-courses of the two binocular summation neurons. (D) Winner-take-all (WTA) index. The conventional model shows dichoptic grating rivalry that is only slightly stronger than plaid component rivalry.

To understand the relationship between rivalry and cross-orientation suppression, we first implemented a firing rate model of binocular rivalry that incorporated normalization (Figure 2A).

The model consisted of two left-eye, monocular neurons selective for orthogonal orientations, two analogous right-eye, monocular neurons, and two binocular summation neurons that received feedforward input from the monocular neurons. While we refer to the units as “neurons” for simplicity, the biological implementation of each unit may be more accurately described as an ensemble of (e.g., 50–100) neurons with similar response properties [30]. The monocular neurons mutually inhibited one another, and the binocular summation neurons mutually inhibited one another, yielding competition at both the monocular and binocular levels. We included inhibitory connections not only between neurons in different eyes, but also between neurons in the same eye. This arrangement differs from the many conventional models that only allow competition between neurons in different eyes, but similar results supporting the same conclusions were found to hold from the conventional model even when competition was restricted to neurons in different eyes.

Mutual inhibition was implemented by the divisive normalization equation [19], [20] rather than by subtraction, which is typically used in models of rivalry. The normalization equation is not intended to be a mechanistic model of suppression, but instead provides a good description of the computations underlying inhibitory interactions in cortex. Biophysically plausible implementations of this equation have been described elsewhere [19], [31]. Lowpass-filtered noise was included in the inputs to each neuron (see *Methods* for details). Unfiltered white noise coupled with neural adaptation would have behaved similarly.

Binocular rivalry occurred in response to dichoptic gratings when the mutual inhibition in the model was set to be strong (by assigning high values to certain weights in the denominator of the normalization equation). Rivalry strength was quantified with a winner-take-all (WTA) index defined in terms of the responses of the binocular summation neurons (see Methods for details). The index was bounded by 0 and 1, where 0 indicated that the two binocular summation neurons always had identical responses, and 1 indicated complete rivalry, with only one or the other neuron exhibiting a non-zero response at each time.

Although strong inhibition permitted rivalry in the dichoptic grating condition, it had the unintended consequence of generating strong rivalry between plaid components (Figure 2B–D). Rivalry occurred for both monocular plaids (orthogonal gratings superimposed in one eye) and for binocular plaids (orthogonal gratings superimposed in both eyes). While the model's plaid component rivalry could be made to be slightly weaker than the dichoptic grating rivalry (Figure 2B–D), actual plaid rivalry in psychophysical experiments is much weaker than the rivalry between dichoptic gratings [15], [16].

An intuition for this behavior can be obtained by considering situations in which the competition between features is either entirely in the binocular stage or entirely in the monocular stage. If the competition is in the binocular stage, dichoptic gratings can easily be made to rival. However, plaids will provide the same input to the binocular stage as dichoptic gratings, and thus their components will rival as well. Alternatively, if the competition is in the monocular stage, a similar problem emerges. The competition that allows dichoptic gratings to rival will also cause rivalry in the components of plaids. One might think that rivalry could be restricted to dichoptic gratings if competition is made to be only interocular, not intraocular. However, while this arrangement will prevent rivalry for monocular plaids, it will allow rivalry in binocular plaids because each component in each eye will compete with an orthogonal component in the other eye.

**Figure 3:**
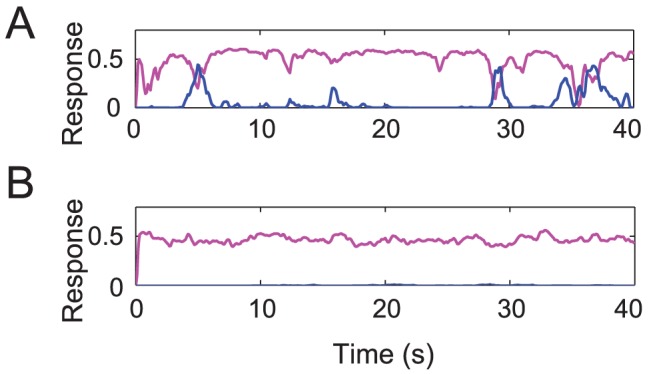
Reliability of simulated binocular layer responses to monocular gratings. (A) Example responses to a monocular grating for one of the 6 parameterizations of the conventional model that passed the initial two criteria (see *Methods*). Magenta, simulated response time-course for a neuron tuned to the orientation of the monocular grating. Cyan, simulated response time-course for a neuron with orthogonal orientation preference. While this parameterization of the model produced stronger rivalry for dichoptic gratings compared to plaids, the model behaved implausibly when presented with a monocular grating. Specifically, the model occasionally showed stronger responses for the non-presented orthogonal grating. (B) Simulated responses of the opponency model were stable, and did not any show any switches in dominance.

An exhaustive grid search through plausible weight values and noise amplitudes did not find a single parameterization that produced reasonable responses to plaids and gratings. While there were a few parameterizations that produced stronger rivalry for dichoptic gratings compared to plaids, all of these parameterizations had such high noise amplitudes and weight values that they behaved implausibly during the presentation of monocular gratings, with responses so volatile that they occasionally responded more strongly to a non-presented orthogonal orientation than to the grating that was presented (see *Methods* and Figure 3). If these models were correct, human observers viewing a single grating monocularly would occasionally perceive an orthogonal grating instead.

**Figure 4:**
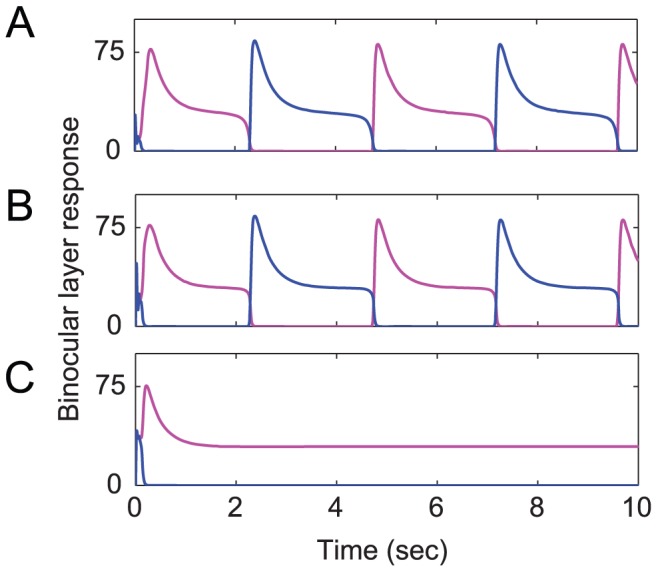
Demonstration that a previously published model (Wilson, 2003) does not show weaker rivalry for binocular plaids and monocular plaids. (A) Standard rivalry under dichoptic conditions. Our simulation results are identical to those of Figure 2A in Wilson (2003). (B) Binocular plaid conditions result in full rivalry. (C) Monocular plaid conditions results in sustained dominance.

We confirmed that a standard model of binocular rivalry [11], with subtractive instead of divisive inhibition, also failed to solve this problem. This model appropriately exhibited strong rivalry for dichoptic plaids, but it also inappropriately exhibited equally strong rivalry with periods of complete dominance for binocular plaids, and sustained dominance with no alternations for monocular plaids (Figure 4). Although we did not explicitly test every previous model of binocular rivalry, we infer that they likewise would exhibit the same problem because none of them include a mechanism to modulate the strength of inhibition depending on whether the two component gratings are dichoptic, binocular, or monocular [9].

### Opponency model

**Figure 5:**
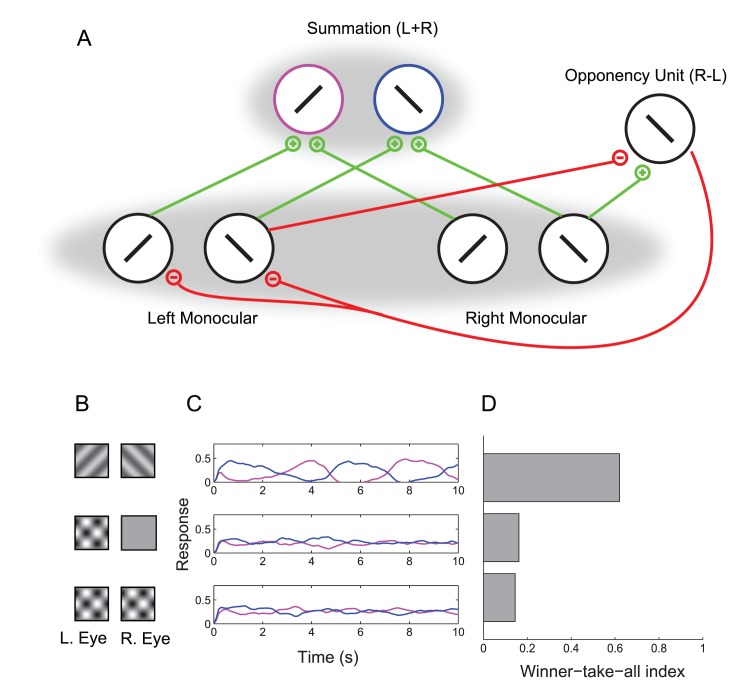
Ocular opponency model. (A) Schematic. An opponency neuron computes a response difference between the two eyes for a particular orientation preference and retinotopic location. The opponency neuron inhibits activity in the opposite eye (curved red line), thus amplifying the winner-take-all behavior of normalization (gray shadow). Not shown are three other opponency neurons (a R-L neuron selective for the orthogonal orientation, and two L-R neurons). Also not shown are the two normalization pools for the opponency neurons (one for R-L opponency neurons, and another for L-R opponency neurons). (B–D) Model simulations (same format as Figure 2). The opponency model shows dichoptic grating rivalry that is much stronger than plaid component rivalry.

A robust solution to the plaid problem was obtained by adding ocular opponency neurons to the conventional model. Each opponency neuron computed a difference between the responses of two monocular neurons corresponding to the same orientation but different eyes (Figure 5A). This difference was then halfwave rectified (setting negative values to zero) and normalized (see Methods for details). Through feedback, the opponency neurons linearly inhibited (i.e., via subtraction) both monocular neurons corresponding to the eye from which they received inhibition. There were a total of 4 opponency neurons, so that both orientations and both differences (right-left and left-right) were included in the model, although only one is shown in Figure 5A.

Rivalry was more than three times as strong for dichoptic gratings compared to plaids (Figure 5B–D). As with the conventional model, we again quantified the strength of rivalry with a WTA index defined in terms of the responses of the binocular summation neurons. Unlike in even the best parameterizations of the conventional model, there were periods of near complete dominance for dichoptic gratings but not plaids, without requiring implausible responses to monocular gratings presented alone. The model accomplished this using fewer parameters than the conventional model (Tables 1 and 2).

**Table 1:** Ocular opponency model parameters.

Parameter	Value	Description
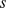	0.5	Semisaturation constant for monocular and summation neurons
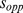	0.9	Semisaturation constant for opponency neurons
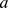	0.05	Noise amplitude (SD of Gaussian white noise)
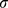	800 ms	Noise smoothness (SD of Gaussian temporal filter)
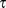	50 ms	Time constant

The model was robust enough that all connection weights were set to 1.

**Table 2:** Parameter values used in the conventional model grid search.

Parameter	Candidate values
Monocular normalization weight: Self-connection	0.4, 0.8, 1.2, 1.6, 2
Monocular normalization weight: Same eye, orthogonal orientation	0.4, 0.8, 1.2, 1.6, 2
Monocular normalization weight: Opposite eye, same orientation	0.4, 0.8, 1.2, 1.6, 2
Monocular normalization weight: Opposite eye, orthogonal orientation	0.4, 0.8, 1.2, 1.6, 2
Summation layer normalization weight: Same orientation	0.4, 0.8, 1.2, 1.6, 2
Summation layer normalization weight: Orthogonal orientation	0.4, 0.8, 1.2, 1.6, 2
Feedforward weights	0.4, 0.8, 1.2, 1.6, 2
Noise amplitude ( 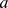 )	0.01, 0.03, 0.05, 0.09, 0.13
Semisaturation constant ( 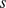 )	0.5
Noise smoothness ( 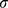 )	800 ms
Time constant ( 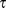 )	50 ms

Ocular opponency neurons solved the plaid problem by responding during conditions in which binocular rivalry might occur. When presented with binocular plaids, the opponency neurons were silent. Under these circumstances, weak cross-orientation suppression occurred because of normalization. In contrast, when dichoptic gratings were presented, the opponency neurons became active and suppressed activity in monocular neurons corresponding to the opposite eye. This suppression amplified the normalization-based competition between the eyes.

It was critical that the opponency neurons inhibited monocular neurons they received inhibition from, rather than exciting monocular neurons they received excitation from. While the latter arrangement encouraged rivalry in dichoptic gratings and not in binocular plaids, it created rivalry (inappropriately) in monocular plaids, because monocular plaids excited the opponency neurons. In the correct arrangement, monocular plaids excited the opponency neurons, but the inhibitory feedback had a negligible effect, because the unstimulated monocular neurons were already responding only very weakly.

### Empirical test of the models

The simulation results demonstrated how an opponency model, but not a conventional model, can exhibit both rivalry and cross-orientation suppression under appropriate circumstances. Nevertheless, the simulation results only showed how it is theoretically possible that opponency cells contribute to rivalry; they did not provide evidence that opponency models are necessary for rivalry. We therefore designed an experiment, using adaptation, to test a prediction of the opponency model. Adaptation is a powerful psychophysical tool, because it supports inferences about selectivity [32], [33], in this case, selectivity for ocular opponency.

**Figure 6:**
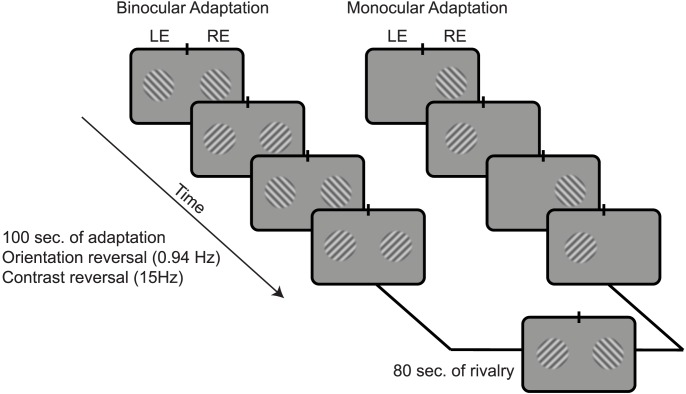
Schematic of a block from the psychophysics experiment. In the binocular adaptation condition (left sequence), the gratings were shown to both eyes. In the monocular adaptation condition (right sequence), the gratings were shown to only one eye at a time, alternating at 0.94 Hz. After the adaptation period, observers viewed orthogonal (rival) stimuli in each eye for 80 sec and reported their percepts with button presses.

Human observers participated in two experimental sessions with different adaptors. During one session, observers adapted to orientation-alternating grating stimuli presented binocularly prior to rivalry (Figure 6; see *Methods*). According to both the conventional model and the opponency model, these stimuli should activate, and therefore adapt, the monocular neurons and binocular neurons, but not the opponency neurons. During the other session, observers adapted to orientation-alternating stimuli presented monocularly, where one orientation was always presented to the left eye and the orthogonal orientation was always presented to the right eye, with only one or the other orientation presented at a time. According to both models, these stimuli should activate, and therefore adapt, the monocular neurons and the binocular neurons. The critical difference between the two models is that according to the opponency model (but not the conventional model), the monocular adaptors will also adapt the opponency neurons.

Following adaptation, observers viewed rival stimuli, with one orientation presented to one eye and the other orientation to the other eye. The rival stimuli were dichoptic gratings, identical in both sessions. Observers reported their percepts with button presses. Intuitively, the opponency model predicts that the monocular adaptation condition should result in weaker rivalry than the binocular adaptation condition, because the monocular condition adapts the opponency neurons that amplify the suppression, resulting in rivalry. The conventional model, which lacks opponency neurons, does not make this prediction.

**Figure 7:**
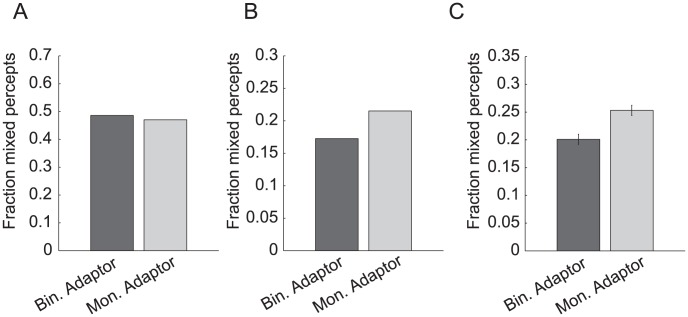
Model predictions and psychophysical results from the adaptation experiment. (A) Conventional model predictions. (B) Opponency model predictions. (C) Psychophysical results. Error bars are the standard error for repeated measures. (Standard error after each observer's mixed percept fractions were recentered to the mean across observers and conditions.)

To make the predictions explicit, we first ran simulations on the two models after adaptation to the two conditions. A long-term adaptation variable (time constant = 80 sec) was added to both models to capture the slow buildup of adaptation produced by our experimental manipulations (see *Methods*). This type of adaptation is slower than the adaptation that is sometimes used in other models of binocular rivalry to capture percept alternations. We could not directly measure the WTA index in human participants, so we relied instead on the percentage of “mixed” percepts as a proxy measure, where a high percentage of mixed percepts corresponds to a low WTA index. The conventional model, which lacks opponency neurons, predicts that monocular adaptation will result in a slightly lower percentage of mixed perception during rivalry compared to binocular adaptation (Figure 7A; *see* *Methods*). In contrast, the opponency model predicts that monocular adaption will result in a higher percentage of mixed percepts compared to binocular adaptation (Figure 7B).

Psychophysical tests on human participants supported the opponency model. We found a higher percentage of mixed perception following monocular adaptation (*M* = 25.3, *SD* = 19.1) compared to binocular adaptation (*M = *20.1, *SD* = 18.4; paired *t*(29) = 2.9, *p*<.01; Figure 7C–D). These results suggest that opponency neurons contribute to rivalry.

Previous research has found that contrast adaptation alone can decrease dominance durations [34]. However, the difference in mixed perception between conditions in our experiment cannot be explained by this mechanism, as overall contrast adaptation would be expected to be higher in the binocular adaptation condition than the monocular adaptation condition. Indeed, the conventional model predicts slightly more mixed perception after binocular adaptation compared to monocular adaptation (Figure 7A), contrary to what we found.

The different adaptor conditions did not cause different eye imbalances. We defined eye imbalance as the absolute difference in the fraction of time that the left eye and right eye delivered the dominant percept. The mean absolute eye imbalance was 0.15 after monocular adaptation, and 0.16 after binocular adaptation. This difference was not significant. (*t*(29) = 0.31, *p*>.05).

The effect of our adaptation manipulation on mixed perception, while significant, was not very large. Small effects are common in adaptation experiments [35]–[37], presumably because the neurons are only partially adapted, not completely silenced. Indeed, our own model simulations predict small effects for this reason. Partial adaptation was a particularly important issue in our experiment, because the opponency neurons were adapted not only by the monocular adaptors, but also by the subsequent rival stimuli that were presented in both conditions. Thus, it was expected that the opponency adaptation condition would generate only marginally more opponency adaptation than the binocular adaptation condition.

## Discussion

We developed a computational model that resolves a tension between theories of binocular rivalry and cross-orientation suppression. Once specified, we used the model to make novel predictions about the effects of adaptation prior to rivalry. Whereas conventional models predict that adaptation to monocular gratings, compared to binocular gratings, will cause a small decrease in mixed perception during rivalry, the opponency model predicts an increase in mixed perception. The opponency model makes this prediction because monocular stimuli uniquely adapt the opponency neurons that amplify the suppression, resulting in rivalry. Psychophysical tests confirmed the predictions of the model, with subjects reporting more mixed perception following adaptation to monocular gratings than binocular gratings.

### Models of binocular rivalry

Conventional models of binocular rivalry rely on strong competition, between neurons tuned to orthogonal orientations, to generate rivalry between dichoptic gratings. Because of this strong competition, conventional models make the incorrect prediction that plaid components, which are also orthogonally oriented, will strongly rival. This problem cannot be solved by adjusting the connection weights between neurons, as demonstrated by an exhaustive parameter search. Lowering the inhibitory weights reduces rivalry for plaids, but also reduces it for dichoptic gratings.

Using ocular opponency neurons, we developed a model of binocular rivalry that solves the plaid problem much more effectively than a conventional model, despite using fewer parameters. The new opponency model makes a clear and novel connection between two of the most well-studied forms of competition in visual cortex: binocular rivalry and cross-orientation suppression. The model also makes predictions about functional interactions between monocular neurons, binocular summation neurons, and ocular opponency neurons. Under our interpretation, binocular rivalry and cross-orientation suppression rely on the same neural computations: orientation-selectivity, rectification, and normalization. In binocular rivalry, however, competition is amplified by feedback from ocular opponency neurons.

A number of published dynamical systems models have characterized the alternating periods of perceptual dominance for dichoptic gratings, but none has provided simulations showing weak rivalry in plaid components in the same model. One published model proposed separate mechanisms for interocular and intraocular suppression, but it is not clear how this type of model could avoid rivalry in binocular plaids, where both interocular and intraocular mechanisms may contribute to suppression [38]. Another published report showed how rivalry occurred in a strong-inhibition variant of a model, whereas weak suppression occurred in a low-inhibition variant of the model, but it was not explained how the inhibition strengths were controlled depending on whether plaids or dichoptic gratings were viewed [39]. Using opponency neurons, our model demonstrates the appropriate behavior for both types of stimuli with a fixed set of parameters. Opponency neurons may be the neurobiological analogue of rivalry XOR (exclusive-OR) units. These XOR units were proposed 23 years ago in a qualitative model of binocular rivalry [28], but the need for this kind of computation has been overlooked ever since.

### Empirical support for ocular opponency

Neurophysiological studies have identified opponency neurons that algebraically subtract the input between the two eyes [21]–[23], but these neurons have received little attention, in large part because the functional significance of these cells was unknown. Livingstone and Hubel (1984) remarked that they were “at a loss to imagine any plausible benefit” for the subtraction operation between eyes. Subsequent theoretical work provided one possible benefit, that opponency neurons may play a critical role in efficient stereo coding [26].

Previous psychophysical studies have provided evidence for ocular opponency, but none have tested the role of opponency in binocular rivalry [24], [25]. Of most relevance is the observation that the amount of mixed perception increases over the course of binocular rivalry due to long-term adaptation during rivalry, and that this effect is not simply due to contrast adaptation [40]. While this effect has been attributed to adaptation of a generic “rivalry mechanism”, these results are easily explained under our framework. During the presentation of rival stimuli, as opponency neurons become increasingly adapted, they become unable to effectively enforce rivalry. Greater mixed perception following adaption was later replicated both with binocular adaptors and monocular adaptors [29]. This latter experiment was very similar to our own, and thus provides additional support for our model. However, it was also observed that when subjects were deprived of stimulation for an hour after binocular adaptation, subsequent mixed perception was still strong. This suggests that long-term plasticity, and not just adaptation, may have been involved. This form of plasticity is not present in the current version of our model. The authors of that study attributed their results to “anti-Hebbian” learning, in which inhibitory connections are weakened following stimulation by monocular or dichoptic stimuli. In the context of our model, anti-Hebbian learning could be incorporated into the inhibitory connections between opponency neurons and monocular neurons, thus providing a mechanism for long-term plasticity.

### Normative accounts of opponency feedback

We do not commit to any normative account for why the visual system would develop the circuitry used in our model, and it remains an open question. No feedback was included in previous models of efficient stereo coding [26]. Instead, gain control was applied to output of the summation and opponency channels to optimize their sensitivities, with stronger gain on the opponency signal. The feedback in our model, which has the effect of increasing the opponency signal more than the summation signal, may be one mechanism by which this gain control is accomplished. Alternatively, some researchers have proposed that binocular rivalry may be a rational form of Bayesian inference, where sampling from the two eyes is used to approximate a posterior distribution over causes [41]. Under this interpretation, the opponency mechanism might be required to allow rivalry only when it is rational (i.e. under dichoptic conditions).

### Model limitations

We made no attempt to account for all of the known properties of binocular rivalry. Instead, we focused on what has been an under-appreciated shortcoming of binocular rivalry theories. For simplicity, our model uses only a few parameters to address that shortcoming. We believe that some of the remaining properties of binocular rivalry could be accounted for by straightforward extensions to our model.

While we included long-term adaptation to simulate our adaptation experiment, we did not include any fast adaptation dynamics, a process that almost certainly plays a role in perceptual alternations during binocular rivalry. Models that incorporate adaptation can account for the gamma distribution of dominance durations and the observation that changing the contrast of one eye primarily affects only the dominance durations of the other eye [42], although the generality of this observation has been called into question [43], [44].

There has been considerable debate about whether binocular rivalry occurs primarily between monocular representations (eye rivalry) or between binocular and higher level representations (image rivalry) [3]. Psychophysical evidence for eye rivalry includes the observations that swapping the images between eyes at the peak of a dominance phase causes an immediate change in perception [28], [45], and that target probes presented to the suppressed eye are difficult to detect [46], [47]. On the other hand, there are two main lines of psychophysical evidence that image rivalry may contribute as well. First, research on interocular grouping has shown that when component patches of two different images are distributed between the eyes, observers often see coherent images [48]. Second, when orthogonal gratings are rapidly swapped between the eyes and accompanied by an even faster flicker, observers report rivalry at a frequency much slower than the swap rate [49]. There is also conflicting physiological evidence over whether rivalry occurs primarily at the monocular level or at later stages [50]–[53].

Our model is hierarchical by design, and thus includes both monocular competition (contributing to eye rivalry) and binocular competition (contributing to image rivalry). Our model is agnostic about whether the binocular neurons underlying perceptual judgments reside in V1 or higher cortical areas. To account for the interocular grouping effects [48], an extension to our model could include top-down modulation of local competition, analogous to computational theories of attention [54], such that portions of one eye's view and complementary portions of the other eye's view are simultaneously dominant [55].

Our current model can only partially account for the observations from rapid swap experiments [49], [56]. The binocular summation neurons in the model exhibit slow alternations during high-frequency alternations between stimuli, but the responses of these neurons are weak under these conditions, and do not show all the known frequency-dependent effects of eye swapping [56]. A previously published hierarchical model used relatively strong inhibition at the binocular layer to produce slow and robust alternations during rapid swap stimulation but, like our model, did not attempt to account for the frequency-dependence [11]. In both models, increasing the inhibition in the binocular layer could shift the behavior more to image rivalry during rapid swap stimulation, but would also increase the rivalry between plaid components.

Finally, our model makes no attempt to explain ‘rivalry memory’, although the extensions to our model would be straightforward. In rivalry memory experiments, the rival stimuli are turned off for several seconds immediately after one of them has become dominant. When the stimuli are turned back on, the previously dominant stimulus is typically perceived [57]–[59]. This effect could be explained by including brief, recurrent synaptic facilitation in our model [60].

## Methods

### Conventional model: Mathematical details

Within each subpopulation of neurons (monocular, binocular summation), mutual inhibition was implemented by a dynamical variant of the normalization equation:
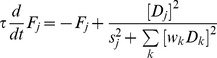
(1)where the brackets indicate halfwave-rectification. At steady state, the instantaneous firing rate 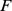 of neuron 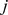 was the half-squared drive of the neuron 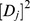 divided by a weighted sum of the half-squared drives of all the other neurons in the normalization pool, plus an additional semi-saturation constant 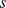 in the denominator.

All four monocular neurons were part of a single normalization pool (Figure 2A). Thus, every monocular neuron contributed to the normalization of every other monocular neuron, including itself. For the binocular summation neurons, the pool consisted of both summation neurons.

The unnormalized drive 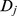 for each monocular neuron was determined as follows:
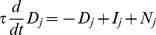
(2)where 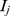 was the stimulus contrast corresponding to the particular eye and orientation represented by neuron 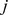. Lowpass filtered noise 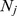 was added to each neuron's input, computed by starting with Gaussian white noise and convolving in time with a Gaussian kernel (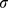 = 800 ms). The noise was statistically independent for each neuron.

The unnormalized drive for each binocular summation neuron depended on excitatory inputs from iso-oriented monocular neurons:
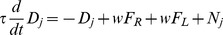
(3)where 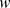 was the feedforward weight, and 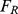 and 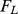 were the activities of the right and left monocular neurons with the appropriate orientation preference.

### Opponency model: Mathematical details

The drive of a right-minus-left (RL) opponency neuron was computed as
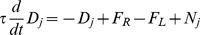
(4)where 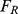 and 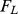 are the activities of the right and left monocular neurons with the appropriate orientation preference. The weights on the feedforward connections were not included in this equation because they were set to 1. In fact, the opponency model was robust enough that we could discard the weight parameters (the 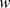
's in equations 1 and 3) setting them all to 1 (Table 1). The RL opponency neurons subtracted left monocular activity from right monocular activity for a particular orientation (Figure 5A, Equation 4). There are a total of 4 opponency neurons, for two orientations and two differences (RL and LR).

The two RL opponency neurons formed a normalization pool separate from the normalization pool for the two LR opponency neurons. Because only a single RL opponency neuron is shown in Figure 5A, the pools are not shown either.

Through feedback, the opponency neurons linearly inhibited both monocular neurons in the opposite eye. Thus, the drive for a left eye monocular neuron tuned to orientation ‘A’ was:
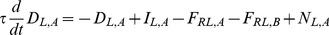
(5)where 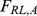 and 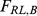 were the firing rates of the two opponency neurons driven by the right eye.

### Numerical simulations

The models were numerically approximated with Euler's Method, using a 2 ms time step. During the grid search and the adaptation simulations, we used a 10 ms time step because of the extreme computational time demands. We tested the conventional model and the opponency model on five conditions: dichoptic gratings, monocular plaids, binocular plaids, monocular gratings, and binocular gratings. Grating contrasts were set to 0.5. Matlab code for the conventional model and the opponency model will be available on our website (http://www.cns.nyu.edu/heegerlab/).

### Winner-take-all index

To determine the extent of rivalry between components, we defined a winner-take-all (WTA) index on the responses of the binocular summation neurons:
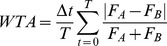
(6)where A and B refer to the two orientations, 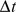 is the time step, and 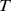 is the simulation duration. The index was bounded by 0 and 1, where 0 indicated that the two binocular summation neurons always had identical responses, and 1 indicated complete rivalry (with only one or the other neuron exhibiting a non-zero response at each time). Reliable estimates of the WTA index were obtained by averaging over 160 seconds of model time (Figures 2 and 5).

### Conventional model grid search

To test whether the conventional model could effectively solve the plaid problem, we performed an exhaustive grid search through plausible parameter values (Table 2). In total, 390,625 parameter combinations were simulated.

For each parameter combination, we simulated the model for 40 seconds in model time and considered the model to be a possible candidate for acceptance if it met two initial criteria described below. Because each simulation of each stimulus condition was accompanied by randomly and independently generated noise, a small number of the 390,625 parameter combinations met our criteria for model success simply by chance. Therefore, using a longer 400 second simulation duration, we repeated the simulations for the models that passed the criterion the first time. The two initial criteria were:

1. A WTA index during dichoptic grating presentation of greater than 0.4.
2. A dichoptic grating WTA index that was at least 60% higher than the WTA index for monocular plaids and binocular plaids.

Of the 390,625 parameter combinations, only 6 met the initial two criteria both times. However, all 6 of these combinations produced implausible behavior during the presentation of monocular gratings: the responses were so volatile that the model would sometimes show stronger responses in the neurons tuned to the orthogonal orientation that was not presented (Figure 3). If these models were correct, human observers presented with a single grating monocularly would occasionally perceive an orthogonal grating instead. We rejected all 6 parameter combinations that passed the first 2 criteria because they all produced multiple percepts switches in the second round of simulations.

Our grid search sampled weight parameters at intervals of 0.4 units and noise amplitudes at intervals of .02 and 0.04 units (Table 2). We cannot rule out the possibility that a parameter combination overlooked by our sampling rule could produce good behavior in the conventional model. Such a parameter combination, if it were to exist, would have to be very finely tuned.

### Psychophysics

Thirty observers (19 females) participated in the psychophysics experiment. All observers had normal or corrected-to-normal vision. All observers were over the age of 18 and provided written informed consent. The experimental protocol was approved by the University Committee on Activities involving Human Subjects at New York University. Two adaptation conditions were conducted in separate sessions on separate days, and the order of sessions was counterbalanced across observers. To control for potential time-of-day effects, each observer participated in each session at roughly the same time of day.

Stimuli were presented on a calibrated CRT display positioned 57 cm from the observer's head. Observers viewed a split screen, through base-out prism glasses, with the left half of the CRT being presented to the left eye and the right half of the screen to the right eye. A black septum blocked contralateral stimuli from reaching the eyes (i.e., so that the left half of the screen was not visible to the right eye and vice versa). Stimuli were composed of grating patches subtending a diameter of 1.2° of visual angle. The contrasts of the gratings were tapered with a raised cosine window (half cycle = 0.3°). To facilitate fusion, stimuli were surrounded by a square black border (side = 2.2°), and a square patch of 1/f image noise was placed above and below each stimulus (side = 3°; position = 4.5° above and below horizontal).

Each session consisted of 6 blocks, each divided into two parts: a 100 sec adaptation period in which observers passively viewed a sequence of stimuli, followed by an 80 sec rivalry period (Figure 6). To further drive the adaptation process prior to rivalry, the last 24 observers viewed two additional 100 sec blocks of the adaptor at the beginning of each session.

In the binocular adaptation session, observers adapted to a contrast-reversing (15 Hz) binocular grating. The grating reversed in orientation at .94 Hz, from 45 degrees clockwise (CW) of vertical to 45 degree counterclockwise (CCW) of vertical (Figure 6). The spatial frequency was 6.6 cycles/° and the contrast was set to 100%. In the monocular adaptation session, the adaptor gratings were identical to those of the binocular adaptation session, except that one eye was stimulated at a time with the CCW grating presented only to the left eye and the CW grating presented only to the right eye (Figure 6).

After the adaptation period of each block, observers performed a traditional binocular rivalry task for 80 sec in which they viewed static gratings (i.e. not contrast-reversing or orientation-alternating). A CCW-of-vertical grating was shown to the left eye and a CW-of-vertical grating was shown to the right eye. To control for individual differences in contrast sensitivity and eye dominance, the contrast of the stimuli were adjusted for each observer and each eye at the beginning of the first session. While the stimuli on the screen remained fixed throughout each block, observers perceived one of the following at any given moment: (1) A dominant CW-of-vertical grating; (2) A dominant CCW-of-vertical grating; (3) A mixed percept, typically appearing as a plaid. Observers reported their percepts by continuously pressing one of three buttons. To minimize individual differences in response criteria, observers were instructed to report a stimulus as dominant only if it comprised 90% or more of their percept. We computed the prevalence of mixed percepts as the overall percentage of time observers reported mixed percepts (out of the total time a button was depressed).

### Model predictions

To capture the effects of adaptation, we added a long-term adaptation variable to each neuron according to
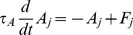
(7)where the time constant of long-term adaptation, 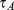, was set to 80 sec. This type of adaptation is slower than the adaptation used to capture perceptual alternations in some conventional models of binocular rivalry. The adaptation variable was then multiplied by a scale factor of 0.5 and subtracted from the unnormalized drive of each neuron.

We simulated 100 blocks of the adaptation experiment using both the conventional model and the opponency model. We presented 100 sec of 100% contrast adaptors that reversed orientation at 0.94 Hz, followed by 80 sec of rival stimuli at 50% contrast. For simplicity, we defined the neurons as invariant to spatial phase, so our stimuli did not reverse in contrast, as in the experiment. Since we could only measure a mixed percept percentage in human observers (rather than a WTA index), we computed an analogous measure for the model simulations. For the 80 sec of rivalry in each block, we first computed a percept index according to
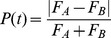
(8)The index was bounded by [0 1], where low values indicated mixed perception and high values indicated dominant perception. Then, to compute the mixed percept fraction, we computed the fraction of time that P(t) was lower than a cutoff of 0.4, and then averaged across all 100 simulation blocks for each model. The pattern of results was robust to variation in the cutoff value.
